# Why, what, and where to publish scientific research

**DOI:** 10.5935/0004-2749.2024-1013

**Published:** 2024-10-08

**Authors:** Newton Kara-Junior

**Affiliations:** 1 Ophthalmology Department, Universidade de São Paulo, São Paulo, SP, Brazil; 2 Editor-in-Chief of Arquivos Brasileiros de Oftalmologia

Scientific researchers have many faces. There are exclusive researchers who work in laboratories or research centers and depend on the government or industry grants; there are those affiliated with specific universities or postgraduate academic programs who conduct research in tandem with their other academic responsibilities; and there are independent researchers with other professional priorities who engage in research when the opportunity arises.

These different types of researchers have different reasons for publishing their work. Those who work exclusively in research should publish in journals with very high scientific impact factors (IF) to ensure continued financial support for their studies. University researchers may publish to ensure the progression of their academic career, or publication may be a requirement of their postgraduate program. Occasional researchers may publish for personal satisfaction, academic or professional status, or to gain the respect of their peers. Thus, publication of one’s research can provide money, resume content, and professional recognition, as well as contributing to and advancing existing knowledge, which is among the objectives of every researcher.

Most university researchers in Brazil, with the assistance of their postgraduate students, demonstrate high levels of methodological rigor in their work. However, the pressure to demonstrate ongoing academic achievement imposed by universities and those responsible for the relevant postgraduate programs can limit researchers in their research choices and oblige them to publish in journals with high IFs. Unfortunately, most of these journals either charge authors high publication fees or charge readers to access articles. Researchers without institutional or government grants to cover such fees often have to pay out of their own pocket, especially if they want their work to be open access^(^1^)^.

Contrary to what many have been led to believe, if one wishes to contribute to the body of existing knowledge and disseminate one’s discoveries among the general population, publishing in high-IF journals is often not the best option. Because the degree of open access offered is lower among high-IF journals, their content may be less accessible than that of less well-known journals.

The term “diamond journals” is used to describe journals that do not charge authors to publish or access articles. These are generally sponsored by field-specific societies and institutions. The scientific IF of diamond journals is gradually increasing, leading to the submission of higher-quality articles and, consequently, further improvement of each journal’s IF. The speed at which this virtuous cycle progresses is increasing, likely in response to the increasingly expensive publication fees of other journals. These are partially attributable to the specialization of the publishing process but are mainly a consequence of greed on the part of international publishers who control many of the most prestigious and widely cited journals^(^2^)^.

The *Arquivos Brasileiros de Oftalmologia* (ABO) is a diamond journal indexed in all major international databases. It offers expedient processing of accepted articles for fast publication, has a good IF, and receives a bonus in the evaluation of national postgraduate programs. In a technical analysis, it is the best alternative to publishing articles from postgraduate theses.

ABO is already well-recognized among international research authors, and we encourage more national re-searchers to publish their main scientific studies with us.
